# Exploring Selenium-Functionalized Hydroxyapatite Using Organic Selenocystine for Antitumor Applications

**DOI:** 10.3390/ma18051043

**Published:** 2025-02-26

**Authors:** Alessandra Barbanente, Anna Maria Di Cosola, Lorenzo Degli Esposti, Michele Iafisco, Mauro Niso, Nicola Margiotta

**Affiliations:** 1 Dipartimento di Chimica, Università degli Studi di Bari Aldo Moro, Via E. Orabona, 4, 70125 Bari, Italy; alessandra.barbanente@uniba.it (A.B.); anna.dicosola@uniba.it (A.M.D.C.); 2 Dipartimento di Chimica e Chimica Industriale, Università degli Studi di Genova, Via Dodecaneso, 31, 16146 Genova, Italy; lorenzo.degliesposti@unige.it; 3 Istituto di Scienza, Tecnologia, e Sostenibilità per lo Sviluppo dei Materiali Ceramici (ISSMC), Consiglio Nazionale delle Ricerche (CNR), Via Granarolo, 64, 48018 Faenza, Italy; michele.iafisco@issmc.cnr.it; 4 Dipartimento di Farmacia-Scienze del Farmaco, Università degli Studi di Bari Aldo Moro, Via E. Orabona, 4, 70125 Bari, Italy

**Keywords:** hydroxyapatite, nanoparticles, selenocystine, selenium, nanomedicine

## Abstract

Selenium (Se) is an essential micronutrient, recognized for its role in cellular redox systems and its therapeutic potential in cancer treatment. Organic selenium compounds, particularly selenocystine (SeCys), have demonstrated anticancer efficacy due to the ability to induce apoptosis and enhance the effects of chemotherapy agents. Recent studies have shown that SeCys exhibits selective toxicity against cancer cells while sparing normal cells. Unfortunately, its clinical application is limited by stability and solubility concerns. A possible solution to overcome these hurdles comes from recent advances in functionalized nanomaterials. In this study, we investigate the possible incorporation of SeCys with hydroxyapatite nanoparticles (HASeCys) via various methods (adsorption, co-precipitation, and co-precipitation through thermal decomplexation), resulting in the formation of nanocomposites with elemental selenium. The highest elemental selenium yield was achieved with a thermal decomplexing co-precipitation, highlighting the influence of synthesis parameters on Se allotrope formation. Finally, as a preliminary investigation, the HASeCys samples were tested on a panel of cancer cell lines, showing an interesting activity when the hydroxyapatite nanocrystals were functionalized with both crystalline gray and amorphous red selenium.

## 1. Introduction

**Scheme 1:**
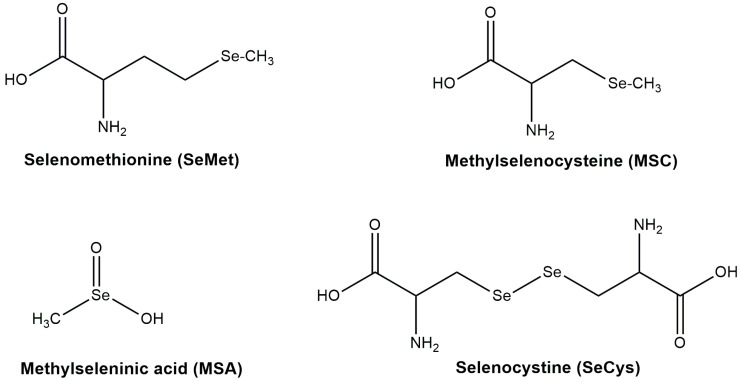
Sketches of selenoamino acids.

Due to its unique biological functions, selenium (Se) is an essential micronutrient for human metabolism, particularly in the oxidation–reduction cellular system [1]. However, 255 μg/day was recently established by the European Food Safety Authority as the tolerable upper intake level for selenium for adult men and women in order to avoid symptoms of chronic selenium toxicity (selenosis) [2]. Se and Se-containing compounds can be grouped into three main categories: inorganic, organic (organo-selenium compounds), and Se-containing nanoparticles (SeNPs) [3,4,5]. In addition to its physiological function, Se is a promising agent for tumor therapy. Organic Se compounds have gained significant interest due to their substantial anticancer effects, often comparable to or even exceeding those of inorganic Se compounds, while exhibiting relatively low toxicity to healthy cells and the organism. The organic Se compounds can be categorized into various families based on their chemical structures, including selenides and diselenides; selenocyanates; derivatives of selenoamino acids, such as selenomethionine (SeMet) and methlyselenocysteine (MSC); methylseleninic acid (MSA; CH_3_SeO_2_H); Se-heterocyclic compounds; and other miscellaneous Se-containing compounds. These organo-Se compounds demonstrate anticancer and chemo-preventive properties through a variety of mechanisms, including the reduction in oxidative stress [6], the induction of apoptosis [7,8,9], and the enhancement of the efficacy of chemotherapeutic agents [10,11,12]. Oxidative stress is generally associated with production of reactive oxygen species (ROS) that can alter cellular DNA, resulting in cell mutations, and also as a consequence of chronic inflammatory processes [13]. The ROS scavenging activities of some Se-proteins such as glutathione peroxidases (GPx), selenoprotein P, and thioredoxin reductases were hence correlated to cellular protective effects of selenium at non-toxic doses. Seleno-amino acid derivatives, such as SeMet and MSC, have been shown to promote apoptosis in solid tumors across various types of human cancer at low concentrations (as low as 0.113 µM). Due to the capacity of organo-Se compounds to induce apoptosis, their synergistic effects with chemotherapeutic agents against cancer have been observed [14]. Among the Se-containing amino acids, selenocystine (SeCys; Scheme 1) is a naturally occurring amino acid [15], while methylselenocysteine (SeMet) and selenocysteine (Sec) are contained in Se-enriched yeast [16]. SeCys is generated because of Sec oxidation [17,18]. This compound exhibits a dual nature of activity—it can act as both an antioxidant and as a prooxidant [17]. It is suggested that its antioxidant activity occurs at nutritional levels [19]. However, SeCys is a compound that exhibits redox activity only after reduction to Sec, which is more nucleophilic than SeCys and thus less stable and more reactive. SeCys has recently garnered significant attention due to its notable anticancer activity and strong selectivity for human cancer cells over normal cells [18]. In vitro studies have demonstrated that SeCys is effective against various cancer types, including human melanoma, cervical, and lung cancer cells [20,21,22]. In combination therapies, SeCys enhances cancer cell death induced by 5-fluorouracil (5-FU) in melanoma cells [23] or by doxorubicin in HepG2 hepatoma cancer cells. Furthermore, the combination of SeCys and auronafin, a thioredoxin reductase (TrxR) inhibitor, amplified the effects of this cytostatic agent, increasing the proportion of apoptotic cells in A549 lung cancer cells [24,25]. SeCys also exhibited potent in vivo anticancer activity in nude xenograft mouse models, significantly inhibiting tumor growth without affecting animal weight [22,26]. Despite demonstrating higher antitumor activity than SeMet, the poor stability and low solubility of SeCys pose significant challenges to its effectiveness and further development as an anticancer drug.

The combination of Se and hydroxyapatite (HA, Ca_10_(PO_4_)_6_(OH)_2_) bioceramic is considered a promising biomaterial for use as a bone scaffold in the treatment of bone tumors and metastasis. HA is highly valued in biomedical engineering due to its excellent biocompatibility and bioactivity and its osteoconductive properties. HA can be synthesized to possess chemical and structural similarity to bone, boosting its bioactivity as bone scaffold in tissue engineering and regenerative medicine [27,28,29]. Recently, we synthesized HA doped with selenite ions, an inorganic form of selenium, for application in bone tumor therapy (HASe). In vitro cell tests indicated that HASe nanoparticles suspension at low selenium concentrations demonstrated good cell cytocompatibility. Conversely, HASe with high selenium concentrations exhibited strong cytotoxic effects on cancer cells but also increased toxicity levels in human bone marrow stem cells (hBMSc) [3,30,31]. In another study, the effect of Se on the osteogenesis properties of HA was investigated using human adipose-derived mesenchymal stem cells (hAD-MSCs), while tests using the human bone osteosarcoma cell line (KHOS-240S) demonstrated the anticancer property of the Se dopant, indicating its potential as a bifunctional scaffold for simultaneous tumor therapy and bone regeneration [32].

**Figure 1:**
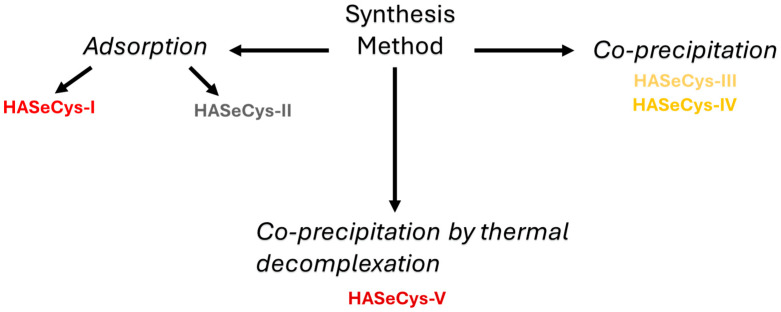
SeCys structure and schematic representation of the methods used to incorporate Se into HA. The different color of the matrices’ names reflects their real color.

Building on the promising effects of SeCys and the potential of HASe for bone tumor therapy, in this work we investigated the use of SeCys as organic Se source to functionalize HA with selenium or SeCys. Several methods were tested, specifically adsorption, co-precipitation, and co-precipitation through thermal decomplexation of calcium ions by citrate (Figure 1). All materials were characterized in terms of chemical composition, morphology, and crystallographic composition. Samples considered most suitable for potential chemotherapeutic application were selected for cytotoxicity evaluation towards a panel of cancer cell lines. Our work shows that HA functionalized with both crystalline gray and amorphous red Se exhibited the highest toxicity in all tumor cell lines and could be used against pancreatic cancer, tumors that are very difficult treat.

## 2. Materials and Methods

### 2.1. Chemicals

High-purity chemical reagents including calcium acetate hydrate (Ca(CH_3_COO)_2_, >99%), orthophosphoric acid (H_3_PO_4_ ≥ 85 wt.% in water), selenocystine (SeCys ≥ 95%), ammonium hydroxide ((NH_4_)OH ≥ 30 wt.% in water), sodium citrate tribasic dihydrate (Na_3_(Cit), HOC(COONa)(CH_2_COONa)_2_·2H_2_O, >99%), calcium chloride (CaCl_2_, >99.99%), sodium carbonate (Na_2_CO_3_, >99.5%), sodium phosphate dibasic (Na_2_HPO_4_, >99%), and nitric acid (HNO_3_ ≥ 70% wt in water) were purchased from Sigma Aldrich (Milan, Italy). Ultrapure water (18.2 MΩ/cm, obtained by a Milli-Q^®^ Direct Water Purification System, Merck Millipore, Darmstadt, Germany) has been used in all the experiments.

### 2.2. Synthesis of HASeCys Nanoparticles

#### 2.2.1. Adsorption Method

SeCys was adsorbed onto lyophilized stoichiometric HA nanoparticles, synthesized according to the method described by Iafisco et al. [33]. Dry HA nanoparticles were suspended in water at 5 mg/mL concentration (30 mg HA nanoparticles in 6 mL) in a 15 mL Falcon tube, to which 3 mg of SeCys was added to achieve a 0.5 mg/mL concentration. The tubes were then vortexed for 15 s and the suspensions were then maintained in a bascule bath at 37 °C and shaken at 60 rpm for 7 days. Subsequently, the suspensions underwent centrifugation (5 min at 2500× *g*). After supernatant removal, the obtained red-colored nanoparticles were washed twice using ultrapure water by centrifugation and redispersion. The material will be referred to as HASeCys-I. The total amount of Se adsorption onto HA nanoparticles was quantified by ICP-OES (Agilent 5100, Agilent Technologies, Santa Clara, CA, USA). The values are presented as mean ± standard deviation (*n* = 3).

The adsorption of SeCys was also carried out using non-stoichiometric, calcium-rich HA nanoparticles, synthesized according to the procedure reported in the literature [34]. In this case, the nanoparticles were not freeze-dried after the synthesis but were used in the suspension form to enhance selenium adsorption. The procedure used for loading SeCys onto non-stoichiometric Ca-rich HA nanoparticles was identical to that reported above for lyophilized stoichiometric HA nanoparticles. The only difference was that in the latter material, the color of the obtained Se-doped nanoparticles was gray. This latter material will be referred to as HASeCys-II.

#### 2.2.2. Co-Precipitation Method

SeCys was incorporated into HA NPs by wet precipitation. A measure of 100 mL of an aqueous solution of Ca(CH_3_COO)_2_ (8.3 mmol) was slowly added (1 drop s^−1^) to 100 mL of an aqueous solution of H_3_PO_4_ (5 mmol) and SeCys (0.3 or 0.6 mmol). SeCys concentration was set at its maximum water solubility (3 mM) or half of the value (1.5 mM), and the resulting materials were referred to as HASeCys-IV (yellow colored) and HASeCys-III (light-yellow colored), respectively. The reaction was carried out at pH = 9 using (NH_4_)OH. The reaction mixture was kept under magnetic stirring at room temperature for 24 h, then the mixture was left standing for about 30 min without stirring to allow the deposition of the inorganic phase. The latter material was isolated by centrifugation of the reaction mixture, repeatedly washed with Milli-Q^®^ water, and freeze-dried at −50 °C overnight.

#### 2.2.3. Co-Precipitation by Thermal Decomplexation Method

The synthesis was carried out by thermal decomplexation of metastable Ca^2+^/citrate complexes in the presence of SeCys, phosphate, and carbonate ions. The synthesis was similar to that reported by Casado et al. [35], with modifications. Two solutions of (a) CaCl_2_ 0.1 M, Na_3_(Cit) 0.4 M and (b) Na_2_HPO_4_ 0.12 M, Na_2_CO_3_ 0.2 M and 0.6 mmol Secys (200 mg) were mixed (1:1 *v*/*v*, 200 mL total) at 4 °C, and the pH was adjusted to 8.5 with diluted HCl. The mixed solutions were introduced in a 250 mL round-bottomed flask, immersed in a water bath at 80 °C and stirred for 24 h. The obtained burgundy precipitate (referred to as HASeCys-V) was subjected to three consecutive cycles of washing by centrifugation with ultrapure water. Afterward, the precipitate was freeze-dried overnight at −50 °C under vacuum.

### 2.3. Characterization of HASeCys

Crystallographic, morphological, and chemical characterization of the materials was performed by powder X-ray diffraction (PXRD), scanning electron microscopy–energy-dispersive spectroscopy (SEM-EDS), and inductively coupled plasma optical emission spectrometry (ICP-OES), respectively.

PXRD patterns were recorded on a D8 Advance diffractometer (Bruker, Karlsruhe, Germany) using Cu Kα radiation generated at 40 kV and 40 mA. PXRD patterns were collected in the 10–60° 2θ range with a step size of 0.02° and a collection time of 0.5 s.

The morphology of the materials was evaluated by field-emission gun scanning electron microscopy (FEG-SEM) (ΣIGMA, ZEISS NTS Gmbh, Oberkochen, Germany) equipped with an EDS probe (INCA Energy 300, Oxford Instruments, Abingdon-on-Thames, UK). Powders were deposited on stubs using adhesive carbon tape and coated with gold in an E5100 Sputter Coater (Polaron Equipment, Watford, Hertfordshire, UK). Micrographs were collected at 5 kV acceleration voltage in secondary electron mode at 5000–100,000× magnifications. EDS compositional analysis was performed with an acceleration voltage of 15 kV. Immediately before sample analysis, the EDS instrument was calibrated by measuring the spectrum of an NIST standard sample of metallic cobalt. Six randomly selected fields were acquired for each material at 2500× magnification.

The Ca, Se, and P content of the materials were measured by ICP-OES using an Agilent 5100 instrument (Agilent Technologies, Santa Clara, CA, USA). A measure of 10 mg of the powdered samples was dissolved in 50 mL of a 1 wt.% HNO_3_ aqueous solution in triplicate. Standard solutions for instrument calibration were prepared from 1000 ppm certified standards (Sigma Aldrich, St. Louis, MO, USA). The values are presented as mean ± 1 standard deviation (*n* = 3).

### 2.4. Biology

All cell culture reagents were purchased from S.I.A.L. S.r.l. (Rome, Italy). MTT (3-[4,5-dimethylthiazol-2-yl]-2,5-diphenyltetrazoliumbromide) was obtained from Sigma-Aldrich (Milan, Italy).

#### 2.4.1. Cell Culture

Human breast adenocarcinoma (MCF7wt), human pancreas adenocarcinoma (PANC-1), human embryonic kidney (HEK-293), and human hepatocellular carcinoma (HepG2) cells were obtained from American Type Culture Collection (ATCC, Bethesda, Rockville, MD, USA). MCF7, PANC-1, and HEK293 were cultured in Dulbecco’s Modified Eagle’s Medium. This medium was supplemented with 10% (*v*/*v*) fetal bovine serum, 1% (*v*/*v*) glutamine, and 1% (*v*/*v*) penicillin–streptomycin. Cells were cultivated at 37 °C with 5% CO_2_ at saturated humidity. HepG2 was cultured in a Minimum Essential Eagle’s Medium. This medium was supplemented with 10% (*v*/*v*) fetal bovine serum, 1% (*v*/*v*) glutamine, 1% (*v*/*v*) penicillin–streptomycin, and 1% non-essential amino acids. Cells were cultivated at 37 °C with 5% CO_2_ at saturated humidity.

#### 2.4.2. In Vitro Cell Viability

The number of living cells was evaluated by the 3-(4,5-dimethylthiazol-2-yl)-2,5-diphenyltetrazolium bromide (MTT) assay [36]. Cells were seeded at a density of 10,000 cells per well into 96-well flat bottom culture plates containing 50 μL of the test samples (ranged from 0.025 mg/mL to 0.2 mg/mL final concentration) in a final volume of 100 μL. Untreated cells were used as positive controls. After 72 h of incubation at 37 °C in a 5% CO_2_ atmosphere, MTT was added to a final concentration of 0.5 mg/mL for a further 3–4 h, the culture medium was removed, and the insoluble product was dissolved by the addition of 100 μL of solvent (1:1 *v*/*v* DMSO/EtOH). The absorbance of each well was measured at 570 nm using a PerkinElmer Victor V3 plate reader. Cell growth inhibition was then calculated as a percentage of cell viability relative to the control and EC_50_ values were determined from dose–response curves by applying a non-linear regression model using GraphPad PRISM version 5.0.

## 3. Results

### 3.1. Synthesis and Characterization of HASeCys

**Figure 2:**
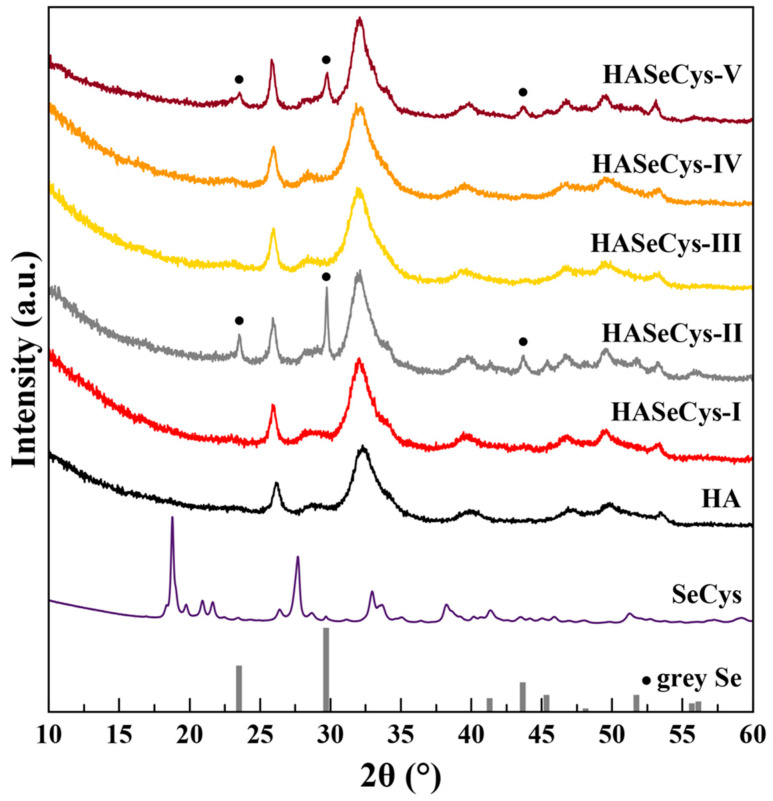
PXRD patterns of HASeCys matrices in comparison to HA, crystalline SeCys, and reference pattern of gray Se. Black dots indicate the diffraction peaks attributed to elemental grey Se.

**Figure 3:**
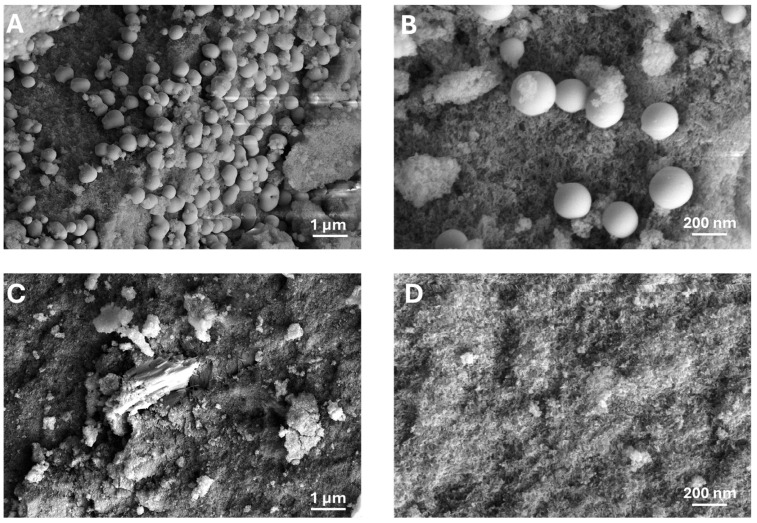
SEM micrographs of HASeCys-I (**A**,**B**) and HASeCys-II (**C**,**D**). The rounded microparticles were attributed to amorphous red Se (**A**,**B**), while the rod-shaped clusters were derived from gray Se (**C**,**D**).

SeCys was used to enrich HA nanoparticles with selenium by adsorption experiments performed at 37 °C in aqueous medium for 7 days using two different types of HA: lyophilized stoichiometric HA and non-lyophilized, non-stoichiometric calcium-rich HA, leading to HASeCys-I and HASeCys-II, respectively. The PXRD pattern of HASeCys-I was in good agreement with reference nanocrystalline HA (Figure 2). The characteristic broad diffraction peaks of HA (2θ = 25.9°, 31.8°, and 39.8°) were evident in the red-colored HASeCys-I, and no peaks ascribable to secondary crystal phases were detected. In particular, the absence of SeCys diffraction peaks (reported for comparison in Figure 2) ruled out the possibility of having obtained a physical mixture of HA and SeCys. On the contrary, the PXRD pattern of the gray-colored HASeCys-II showed, in addition to HA diffraction peaks, other peaks that were attributed to elemental Se (ASTM Card file No. 00-006-0362, reported as stick pattern in Figure 2). Micrographs of HASeCys-I showed nanocrystalline HA nano-platelets approx. 50–100 nm long and 20–40 nm wide, covered by rounded microparticles attributed to red amorphous Se (Figure 3A,B), while FEG-SEM micrographs of HASeCys-II showed rod-shaped clusters derived from Se (Figure 3C,D).

The chemical composition of the materials was analyzed using ICP-OES. The amount of Se (0.34 and 0.39 wt.% for HASeCys-I and HASeCys-II, respectively) and the bulk Ca/(P + Se) ratio (1.54 and 1.59 for HASeCys-I and HASeCys-II, respectively) were comparable between the two materials (Table 1).

**Figure 4:**
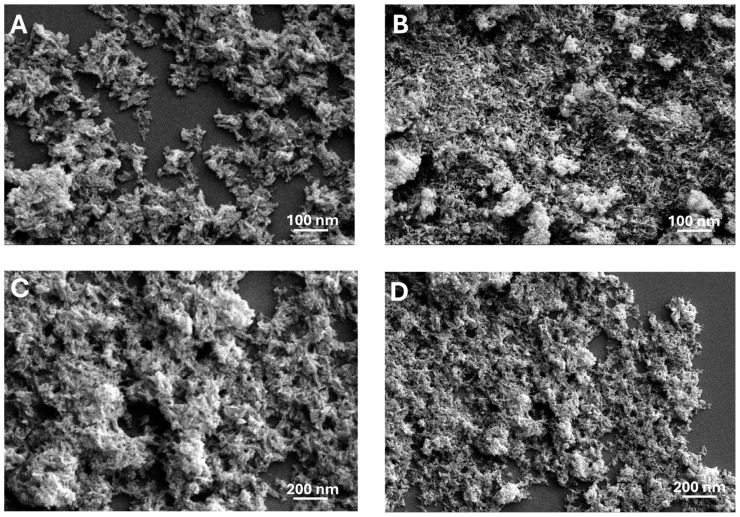
SEM micrographs of HASeCys-III (**A**,**B**) and HASeCys-IV (**C**,**D**).

A second approach to loading SeCys onto HA was co-precipitation synthesis via the slow addition of an aqueous solution of Ca(CH_3_COO)_2_ to an aqueous solution of H_3_PO_4_ and SeCys at pH = 9 and room temperature. HASeCys NPs were synthesized by using two different amounts of SeCys, namely 0.3 (HASeCys-III) and 0.6 mmol (HASeCys-IV). The colors of the matrices obtained were light-yellow and yellow, respectively. The PXRD pattern of HASeCys-III and HASeCys-IV (Figure 2) were ascribable to HA, confirming the achievement of a single phase of nanocrystalline HA. SEM micrographs showed that both samples are composed of nanocrystalline particles, with a platelet morphology characteristic of nanocrystalline HA (Figure 4A–D).

The chemical composition of HASeCys-III and HASeCys-IV (Table 1) confirmed the presence of Se in the samples proportional to the amount used in the co-precipitation, as Se is 0.25 wt.% and 0.51 wt.% for HASeCys-III and HASeCys-IV, respectively. The bulk Ca/(P + Se) ratio was comparable for the two samples (1.48 and 1.50 for HASeCys-III and HASeCys-IV, respectively), while the bulk Se/(P + Se) ratio ranged from about 0.006 in HASeCys-III to 0.012 in HASeCys-IV (Table 1).

**Figure 5:**
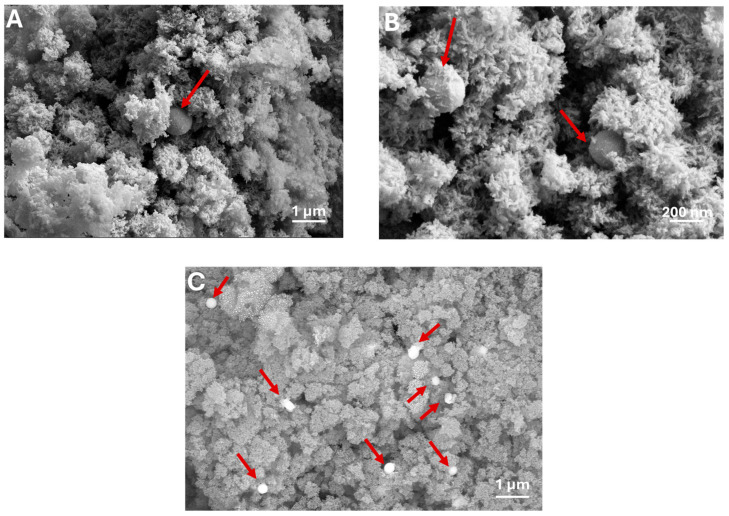
SEM micrographs of HASeCys-V, acquired in secondary electrons mode (**A**,**B**) or in backscatter mode (**C**). The red arrows indicate the rounded microparticles attributed to red amorphous selenium.

Finally, we attempted an alternative co-precipitation method to incorporate SeCys onto HA by using a thermal decomplexation of a metastable citrate/Ca^2+^ solution to control precipitation rate in presence of SeCys, phosphate, and carbonate ions at pH 8.5 and 80 °C. The PXRD pattern of the resulting burgundy-red Se-doped HA sample (HASeCys-V), similar to that of HASeCys-II, exhibited diffraction peaks belonging to nanocrystalline HA as well as crystalline gray elemental Se (Figure 2). FEG-SEM micrographs revealed the presence of spherical Se microparticles (Figure 5A–C). Chemical composition analysis showed that HASeCys-V possess a Se content of 0.91 wt.% with a bulk Ca/P and Se/(P + Se) ratios of 1.52 and 0.027, respectively (Table 1).

**Figure 6:**
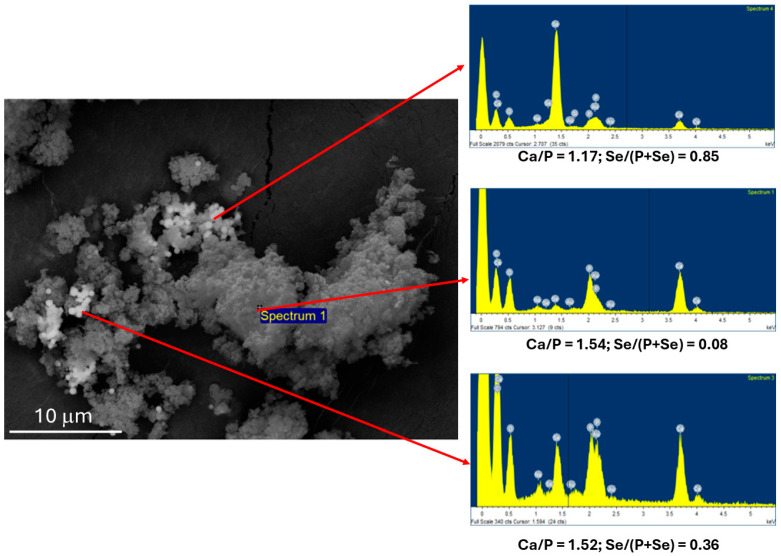
EDS analysis of various regions in the selected FEG-SEM micrograph of HASeCys-V. The red arrows indicate the regions of FEG-SEM micrograph analyzed by EDS.

EDS analysis of the FEG-SEM micrograph (Figure 6) of the nanocrystal regions showed predominantly Ca and P, with limited Se content, and the Ca/P ratio is consistent with the values obtained from ICP-OES analysis of the bulk materials (Table 1). In contrast, the regions containing the spheres exhibit a significantly higher Se content and a lower Ca/P ratio. Although PXRD indicated the presence of crystalline elemental Se, no gray allotropic Se crystals were observed in the FEG-SEM micrographs, unlike in HASeCys-II.

### 3.2. Cytotoxic Assays

**Figure 7:**
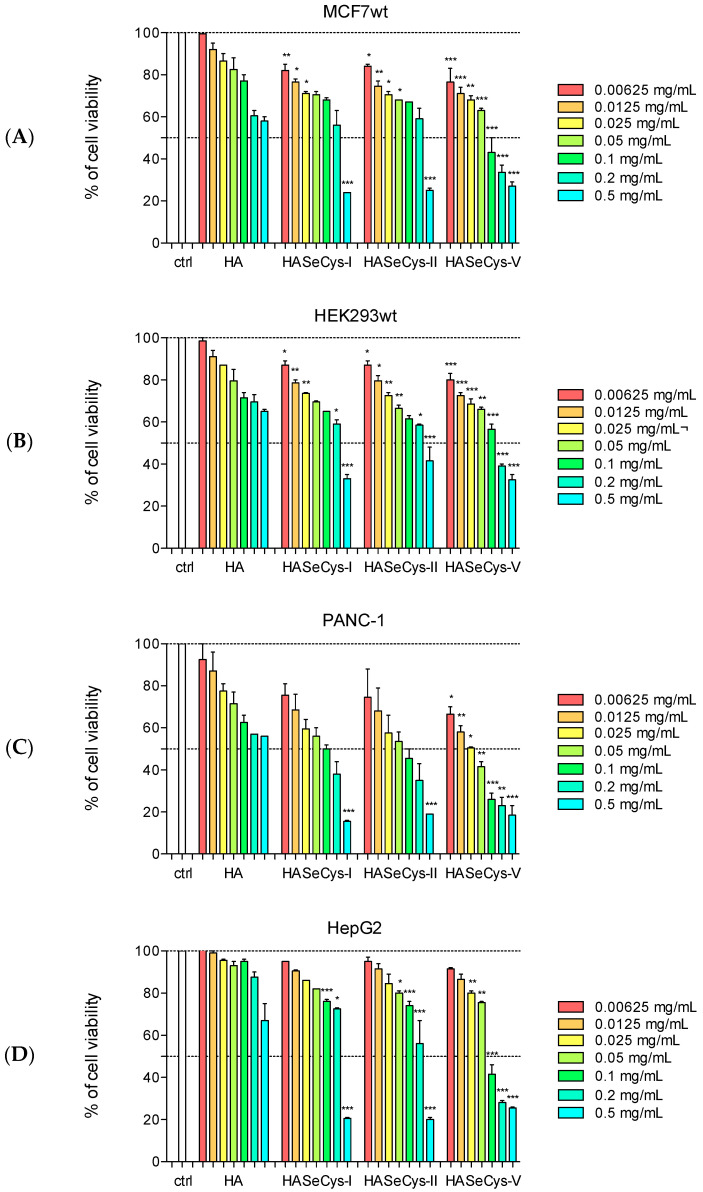
Viability of MCFwt (**A**), HEK293wt (**B**), PANC-1 (**C**), and HepG2 (**D**) after 72 h of exposure time with HA and HASeCys suspensions at different concentrations (0.00625, 0.0125, 0.025, 0.05, 0.1, 0.2 and 0.5 mg/mL). Each bar represents the mean ± SD of three experiments in triplicate: one-way ANOVA followed by Bonferroni’s Multiple Comparison Test * *p* < 0.05, ** *p* < 0.01, *** *p* < 0.001 vs. HA at the same concentration.

The in vitro cytotoxicity of suspensions (0.00625, 0.0125, 0.025, 0.05, 0.1, 0.2 and 0.5 mg/mL) of HA, HASeCys-I, HASeCys-II, and HASeCys-V were evaluated by assessing the viability of MCF7wt, HepG2, PANC-1, and HEK293wt cancer cell lines using the MTT assay (Figure 7). All samples demonstrated a clear dose-dependent cytotoxic effect against all tested cell lines. Notably, HASeCys-I and HASeCys-II exhibited similar moderate toxicity, with less pronounced effects compared to HASeCys-V. Interestingly, in HepG2 cells HASeCys-II (0.2 mg/mL) proved to be more effective than HASeCys-I at the same concentration and comparable Se content, reducing cell viability to approximately 50%, compared to ~70% for HASeCys-I. Notably, PANC-1 cells demonstrated greater sensitivity to HASeCys samples compared to the other tested cell lines, as evidenced by the lowest EC_50_ values (Table 2) determined from dose–response curves for this pancreatic tumor cell line: 0.13, 0.007, 0.003 mg/mL for HASeCys-I, HASeCys-II, and HASeCys-V, respectively. HASeCys-V exhibited the highest toxicity, significantly reducing cell viability even at lower concentrations. Specifically, in MCF7wt and HepG2 cells, HASeCys-V at a concentration of 0.1 mg/mL reduced cell viability to approximately 40%, while in PANC-1 cells, viability was reduced to ~20%. The greatest cytotoxicity of HASeCys-V is evident also by the lowest EC_50_ obtained in all treated cell lines (Table 2): 0.06, 0.03, and 0.07 mg/mL for MCF7wt, PANC-1, and HEpG2, respectively. Finally, immortalized HEK293wt cells were less sensitive to HASeCys-V compared to the cancer cell lines, with significant toxicity observed only at higher concentrations (0.2 and 0.5 mg/mL) and an EC_50_ value of 0.13 mg/mL.

## 4. Discussion

Organo-Se—in the form of SeCys—was used to enrich HA nanoparticles with selenium by different methods (Figure 1). Adsorption experiments were performed using lyophilized stoichiometric HA and non-lyophilized, non-stoichiometric calcium-rich HA. This latter matrix was tested as it possesses a higher specific surface area to maximize SeCys adsorption, as well as a higher concentration of calcium ions, which could facilitate the interaction with the anionic SeCys at basic pH. In fact, it is well-known that dry processes can induce nanoparticle aggregation, leading to a decrease in specific surface area. After incubation, we obtained red HASeCys-I and gray HASeCys-II from stoichiometric HA and Ca^2+^-rich HA, respectively. The PXRD pattern of the red-colored HASeCys-I showed no peaks ascribable to secondary crystal phases, contrary to that of the gray-colored HASeCys-II that showed peaks ascribable to elemental Se (Figure 2). Se can exist in several allotropic forms; red Se is usually an amorphous powder, while gray Se has a crystalline hexagonal structure [37]. Thus, we could assume that in the case of HASeCys-I nanocrystals containing amorphous red Se were obtained, whereas for HASeCys-II, nanoparticles mixed with crystalline gray Se were prepared. These results suggest that during the process, SeCys was reduced to elemental Se in both cases, forming composite materials. To confirm this hypothesis, we analyzed the materials’ morphology using FEG-SEM. Micrographs of HASeCys-I showed nanocrystalline HA nano-platelets covered by amorphous red Se rounded microparticles (Figure 3A,B), while HASeCys-II showed gray Se in the form of rod-shaped clusters (Figure 3C,D). In the literature, the synthesis of red Se allotrope nanoparticles was reported by mixing glutathione (GSH) and Na_2_SeO_3_ in a 4:1 molar ratio, with NaOH added to the mixture to initiate the reaction, specifically in a 5:3 NaOH:GSH molar ratio [38]. The most widely accepted mechanism reports that GSH and Na_2_SeO_3_ mixture forms selenodiglutathione, which is in equilibrium with glutathione disulfide and elemental Se. The addition of NaOH shifts this equilibrium toward elemental Se nucleation [39]. Additionally, red elemental Se, formed in the redox system of selenite and GSH or other reducing agents, is unstable and may further aggregate into gray and black elemental Se depending on conditions such as temperature and the duration of the reaction [40]. Taking this into account, we hypothesize that during incubation with stoichiometric HA the Se-Se bond in SeCys was broken, leading to the reduction in SeCys into red elemental selenium (Se^0^). This transformation is likely favored by the long adsorption period (7 days) and the temperature of 37 °C. In the second procedure, the suspension of calcium-rich HA generates a more basic environment (pH 9) compared to the physiological conditions of the first procedure (pH 7.4). These increased basic conditions may favor the conversion of red allotrope selenium to gray elemental Se [39]. The chemical composition obtained by ICP-OES showed that the amount of Se and the bulk Ca/(P + Se) ratio of HASeCys-I and HASeCys-II were comparable (Table 1), indicating that the amount of Se is independent of the allotrope or nature of HA material.

By the second approach to loading SeCys onto HA, the co-precipitation synthesis, two different amounts of SeCys were used, namely 0.3 (HASeCys-III, light-yellow) and 0.6 mmol (HASeCys-IV, yellow), the latter representing the solubility limit for SeCys in the experimental conditions used. The PXRD patterns of HASeCys-III and HASeCys-IV (Figure 2) showed only a single phase of nanocrystalline HA, ruling out the presence of crystalline SeCys and crystalline elemental Se, which were confirmed also by SEM micrographs, showing HA nanocrystals devoid of amorphous red Se microparticles (Figure 4). These results suggest that co-precipitation did not result in the formation of either red amorphous or crystalline gray selenium secondary phases. This indicates that with this process, the loading of SeCys onto HA occurred as SeCys adsorption onto HA nanocrystals. This outcome may be attributed to the milder reaction conditions used (room temperature, 24 h), which may not be sufficient to induce SeCys degradation and reduction. Moreover, the chemical composition of HASeCys-III and HASeCys-IV (Table 1) showed that the Se inclusion as well as the bulk Se/(P + Se) ratio in both HA crystals is proportional to the amount used in their synthesis, also suggesting that the adsorbed amounts of SeCys did not alter materials’ chemical composition.

As a third attempt to load SeCys onto HA, we adopted a thermal decomplexation of a metastable citrate/Ca^2+^ solution to control the precipitation rate in the presence of phosphate and carbonate ions at pH 8.5 and 80 °C. The resulting burgundy-red Se-doped HASeCys-V was found, in the PXRD pattern, to be composed of nanocrystalline HA as well as crystalline gray elemental Se (Figure 2), similarly to HASeCys-II, while FEG-SEM micrographs revealed the presence of spherical Se microparticles (Figure 5), probably corresponding to amorphous red Se, similar to those observed in the HASeCys-I sample. These results indicate that under the given conditions, SeCys was again reduced to Se^0^, forming both gray and red Se, likely due to the high temperature and basic pH forming a selenium-HA composite material. This process may also be attributed to the milder reducing properties of citrate, which could favor the reduction in SeCys to elemental Se. EDS analysis of the FEG-SEM micrograph confirmed the identification of the spheres as amorphous elemental Se (Figure 6). However, although PXRD indicated the presence of crystalline elemental Se, unlike in HASeCys-II, no gray allotropic Se crystals were observed in the FEG-SEM micrographs. The absence of gray Se crystals in HASeCys-V could be due to their low quantity or small particle size. Interestingly, the ICP-OES analysis indicated that HASeCys-V possess the highest Se content (0.91 wt.%) among all the HASeCys matrices prepared in this work (Table 1), suggesting that thermal decomplexation method is the most effective for Se functionalization.

In order to assess the effect of the composites of HA with different forms of elemental Se, we decided to test the cytotoxicity of HASeCys-I, HASeCys-II, and HASeCys-V. HASeCys-III and HASeCys-IV were not tested since they were not sufficiently stable due to a reduction in the adsorbed amino acid during the time necessary for executing the cytotoxicity tests. The in vitro cytotoxicity of suspensions of HA, HASeCys-I, HASeCys-II, and HASeCys-V was evaluated by assessing the viability of MCF7wt, HepG2, PANC-1, and HEK293wt cancer cell lines (Figure 7), which represent models of human breast, liver, pancreas, and kidney tumors. The less pronounced effects of HASeCys-I and HASeCys-II, compared to HASeCys-V, could be correlated to the highest Se content of the latter’s matrix. As far as the elemental selenium influence on the cytotoxic effect, HASeCys-II functionalized with gray Se proved to be more effective than the red selenium one HASeCys-I at the same concentration and comparable Se content in HepG2 cells. HASeCys-V, functionalized with both crystalline gray and amorphous red Se, exhibited the highest toxicity in all tumor cell lines. Notably, PANC-1 cells demonstrated greater sensitivity to HASeCys samples compared to the other tested cell lines.

## 5. Conclusions

We explored the incorporation of Se with hydroxyapatite using SeCys as an organic-Se source, employing various functionalization methods to leverage the anticancer potential of organo-selenium compounds and the therapeutic properties of Se-functionalized HA for tumor treatment. The functionalization method, reaction time, temperature, and pH influenced the fate of SeCys. Interestingly, attempting adsorption on both lyophilized HA and a Ca^2+^-rich HA suspension, we observed the reduction in SeCys to elemental Se, forming composites with red and gray Se, respectively. This reduction may be influenced by factors such as reaction time, temperature, and solution pH. In fact, with co-precipitation conducted under milder conditions, we did not observe the formation of allotropic Se forms, leading us to hypothesize a SeCys adsorption on HA. Further works may confirm this explanation by using solid-state NMR, although the materials were proven to be unstable. On the contrary, thermal decomplexation of a metastable solution containing Ca^2+^/citrate in presence of SeCys, phosphate, and carbonate ions resulted in the reduction in SeCys to elemental Se, with the highest observed Se content in the composite nanocrystals. Thus, at high temperature and prolonged reaction time, we consistently noted the reduction in SeCys to elemental Se, resulting in both red and gray allotropic forms. It is important to highlight that red allotrope Se nanoparticles have recently been found to exhibit low toxicity toward healthy, non-cancerous cells, such as osteoblasts and human dermal fibroblasts (HDFs) [38,41,42]. Another study demonstrated that red, and to a lesser extent gray, nanosized Se could serve as potential antibacterial coatings to prevent bacterial colonization on PEEK medical devices, inhibiting growth and biofilm formation of Pseudomonas aeruginosa [43]. Given the emerging therapeutic potential of elemental Se, we proceeded to test HASeCys I, II, and V composites against a panel of cancer cell lines. All samples showed a dose-dependent cytotoxic effect, with HASeCys-V, which features both red and gray Se and has the highest Se content, showing the highest cytotoxicity compared to HASeCys-I (red Se) and II (gray Se) across all tested cell lines. In particular, pancreatic cancer cells, which form tumors that are very difficult to treat, demonstrated increased sensitivity to the HASeCys composites. A potential direction for future research could involve investigating the experimental factors that control the preferential formation of one Se allotrope over another in the thermal decomplexing co-precipitation. Building on our interest in Se-functionalized HA bioceramics as promising biomaterials for applications as a bone scaffold in the treatment of bone tumors and metastasis, we intend to explore the potential cytotoxic activity of the tested matrices against this type of tumor and conduct an in-depth investigation into the mechanism of action responsible for the marked activity of HASeCys-V against pancreatic cancer. Finally, since preferential activity towards tumor cells rather than healthy ones is an important goal to achieve in the development of nanomedicines with anti-tumor activity, we intend to carry out selectivity studies using co-cultures of cancer cells and healthy cells.

## Figures and Tables

**Table 1:** Composition of HASeCys samples (calcium, phosphorous and selenium content, bulk Ca/P, bulk Ca/(P + Se) and, bulk Se/(P + Se)). Values are reported as mean value ± 1 SD, averaged out of at least three replicates.

Sample	Ca wt.%	P wt.%	Se wt.%	Ca/P	Ca/(P + Se)	Se/(P + Se)
HASeCys-I	30.0 ± 1.1	14.9 ± 0.5	0.34 ± 0.04	1.55 ± 0.01	1.54 ± 0.01	0.009 ± 0.001
HASeCys-II	29.9 ± 0.7	14.5 ± 0.2	0.39 ± 0.02	1.59 ± 0.01	1.57 ± 0.01	0.010 ± 0.001
HASeCys-III	32.1 ± 0.3	16.6 ± 0.1	0.25 ± 0.01	1.49 ± 0.01	1.48 ± 0.01	0.006 ± 0.001
HASeCys-IV	31.4 ± 1.3	15.9 ± 0.7	0.51 ± 0.02	1.52 ± 0.01	1.50 ± 0.01	0.012 ± 0.01
HASeCys-V	25.3 ± 0.9	12.9 ± 0.4	0.91 ± 0.55	1.51 ± 0.01	1.48 ± 0.02	0.027 ± 0.015

**Table 2:** Antiproliferative activity (EC_50_—half maximal effective concentration—mg/mL) obtained by the MTT test after 72 h of incubation with different concentrations of the tested materials. Data are expressed as mean ± standard deviation (SD) of three independent experiments, each performed in triplicate. MCF7wt—human breast adenocarcinoma cells; PANC-1—human pancreas adenocarcinoma cells; HEK-293—human embryonic kidney cells; HepG2—human hepatocellular carcinoma cells. n.d.—not detected.

Antiproliferative Effect (EC_50_ mg/mL ± SD)				
Samples	MCF7wt	PANC-1	HepG2	HEK293wt
HA	n.d.	n.d.	n.d.	n.d.
HASeCys-I	0.38 ± 0.03	0.13 ± 0.02	0.48 ± 0.06	0.35 ± 0.06
HASeCys-II	0.27 ± 0.03	0.07 ± 0.01	0.27 ± 0.03	0.20 ± 0.04
HASeCys-V	0.06 ± 0.01	0.03 ± 0.01	0.07 ± 0.02	0.13 ± 0.02

## Data Availability

The original contributions presented in this study are included in the article. Further inquiries can be directed to the corresponding authors.
